# Overexpression of MiR-183/96/182 Triggers Retina-Like Fate in Human Bone Marrow-Derived Mesenchymal Stem Cells (hBMSCs) in Culture

**DOI:** 10.1155/2019/2454362

**Published:** 2019-12-11

**Authors:** Mohammad-Reza Mahmoudian-Sani, Fatemeh Forouzanfar, Samira Asgharzade, Nilufar Ghorbani

**Affiliations:** ^1^Thalassemia and Hemoglobinopathy Research Center, Health Research Institute, Ahvaz Jundishapur University of Medical Sciences, Ahvaz, Iran; ^2^Neuroscience Research Center, Mashhad University of Medical Sciences, Mashhad, Iran; ^3^Department of Neuroscience, Faculty of Medicine, Mashhad University of Medical Sciences, Mashhad, Iran; ^4^Cellular and Molecular Research Center, Basic Health Sciences Institute, Shahrekord University of Medical Sciences, Shahrekord, Iran

## Abstract

Retinal degeneration is considered as a condition ensued by different blinding disorders such as retinitis pigmentosa, age-related macular degeneration, and diabetic retinopathy, which can cause loss of photoreceptor cells and also lead to significant vision deficiencies. Although there is no efficient treatment in this domain, transplantation of stem cells has been regarded as a therapeutic approach for retinal degeneration. Thus, the purpose of this study was to analyze the potential of human bone marrow-derived mesenchymal stem cells (hBMSCs) to differentiate into photoreceptor cells via transfection of microRNA (miRNA) in vitro for regenerative medicine purposes. To this end, miR-183/96/182 cluster was transfected into hBMSCs; then, qRT-PCR was performed to measure the expression levels of miR-183/96/182 cluster and some retina-specific neuronal genes such as OTX2, NRL, PKC*α*, and recoverin. CRX and rhodopsin (RHO) levels were also measured through qRT-PCR and immunocytochemistry, and subsequently, cellular change morphology was detected. The findings showed no changes in the morphology of the given cells, and the expression of the neuroretinal genes such as OTX2, NRL, and PKC*α*. Moreover, recoverin was upregulated upon miR-183/-96/-182 overexpression in cultured hBMSCs. Ectopic overexpression of the miR-183 cluster could further increase the expression of CRX and RHO at the messenger RNA (mRNA) and protein levels. Furthermore, the data indicated that the miR-183 cluster could serve as a crucial function in photoreceptor cell differentiation. In fact, miRNAs could be assumed as potential targets to exploit silent neuronal differentiation. Ultimately, it was suggested that in vitro overexpression of miR-183 cluster could trigger reprogramming of the hBMSCs to retinal neuron fate, especially photoreceptor cells.

## 1. Introduction

Degenerations and dystrophies in various neural retina subtypes are known as the major causes of eye disorders leading to blindness [1]. Such conditions are usually characterized by apoptotic death of photoreceptors, and even failure of photoreceptor regeneration has been reported in animals and humans [2]. Thus, few therapeutic strategies have been designed to preserve or restore vision, such as administration of growth factors, laser therapy, and antiangiogenic therapy [3]. Hence, cell replacement has been demonstrated as a potentially therapeutic approach for treating retinal degeneration diseases that possibly aid in restoration of some vision levels [4]. It should be noted that stem cells are undifferentiated cell sources defined by their abilities to self-renew and differentiate into different mature cells [5]. Mature photoreceptors, retinal progenitor cells (RPC), and retinal pigment epitheliums (RPE) are also recognized as sources of adult stem cells used in experimental cell therapy [6–8]. The clinical use of these cell sources can thus bring about some problems including complications of microsurgical procedures, limited number of pluripotent retinal stem cells, differentiation into photoreceptors, formation of an abnormal cell orientation, and rejection by host immune systems [2]. A number of investigations have further shown the multilineage potential of mesenchymal stem cells (MSCs) into neural ones [9]. These findings have also prompted researchers to attempt their induction into photoreceptors for the treatment of degenerative retinal diseases [1]. In this domain, the bone marrow is acknowledged as an ideal source of pluripotent stem cells that has a highly undifferentiated and self-renewing potential. Because of its autologous characteristics, relative ease of isolation, and less controversial nature, this pool of pluripotent stem cells remains as a forerunner of the cells of choice in the treatment of diseases via cell-based therapies [9]. There have been also some reports about differentiation of bone marrow mesenchymal stem cells (BMSCs) into photoreceptors through inducing MSCs cultured with photoreceptors and conditioned medium in rat models [1, 10]. MiRNA-based differentiation is similarly known as a promising alternative technique that does not involve integration into the genome [11]. MiRNAs can also repress mRNA translation or lead to target mRNA degradation via complementarity binding to the 3′-UTR of target mRNAs [12]. MiRNAs are also involved in numerous normal and pathological cellular and physiological processes through regulating the expression of their target genes [12]. Moreover, miRNAs play an important role in maturation and functioning of the mammalian retinas [13]. Recent investigations have further demonstrated that the overexpression of both miR-9 and miR-124, both expressed in postmitotic neurons, induces differentiation of fibroblasts into neurons, and these results have also shown that miRNAs are sufficient to alter cell fate without any transduction of exogenous genes [14]. MiRNA expression patterns are correspondingly associated with photoreceptor differentiation, and photoreceptor-specific miRNAs can be involved in photoreceptor maturation [15]. The miR-183/96/182 cluster (miR-183 cluster) is also highly conserved, intragenic, and sensory organ-specific [16]. It has been found that miR-183 cluster can be abundantly expressed in neurosensory organ and directly target multiple retinal pigmentosa epithelial development-related genes [17]. As well, miR-183 cluster has minimal expression in embryonic retina but it increases after birth and maximal expression in adult retina [17]. MiR-183 cluster also plays an important role in postnatal functional differentiation, morphogenesis, and synaptic connectivity of photoreceptors [16, 18, 19]. In this respect, the knockout of miR-183 cluster or miR-183/96 can even significantly influence progressive retinal degeneration [18, 20]. In the present study, a novel miRNA-based strategy was developed to differentiate hBMSCs into photoreceptor-like cells. It was also found that treatment of mimic-miR-183/96/182 could induce photoreceptor-like cell differentiation in hBMSCs through upregulating orthodenticle homeobox 2 (OTX2) and cone-rod homeobox (CRX), transcription factor neural retina leucine zipper (NRL), and protein kinase C (PKC). Moreover, the BMSC-derived photoreceptor-like cells expressed recoverin and RHO membrane proteins in mature photoreceptors. This study indicated that overexpression of the miR-183/96/182 in hBMSCs was a useful tool for direct photoreceptor differentiation via regulating multiple key photoreceptor-specific genes; thus, it was suggested as a therapeutic potential for retinal degenerative diseases.

## 2. Material and Methods

### 2.1. Primary Cell Culture

MSCs were firstly isolated from the iliac crest of healthy donors based on the protocol of BMSCs harvest procedures approved by competent ethical authorities. The BMSCs were then separated by a density gradient centrifugation and suspended in *α*-MEM containing 20% fetal bovine serum (FBS; Gibco-Invitrogen), 1% L-glutamine (Gibco-Invitrogen), and 1% penicillin/streptomycin (Sigma) followed by plating at an initial seeding density of 1 × 106 cells/cm^2^. After 4 days, the culture medium was changed and the nonadherent cells were removed through washing with PBS, and adherent cells were then cultured until they reached 70%–80% confluence. Subsequently, the cells were subcultured in Dulbecco's Modified Eagle's medium (DMEM; Gibco, Paisley, UK) in low glucose using the same supplement. All the given experiments were carried out using BMSCs from the independent donors at passages 3-4.

### 2.2. Multilineage Differentiation

At this stage, adipogenesis was induced in cultures through addition of differentiating medium, DMEM-LG supplemented with 10% FBS, hydrocortisone (0.5 *μ*M), oboutyl methyl xanthine (0.5 *μ*M), indomethacin (60 *μ*M), and insulin (10 *μ*g/ml), for 21 days. The medium was also changed three times per week. Lipid droplets were then revealed by staining with Oil Red Osteogenic (Oil Red O) induced in cultures via addition of differentiating medium, DMEM-LG supplemented with 10% FBS supplemented with ascorbic acid (2.0′10^−4^ M), *β*-glycero-phosphate (7′10^−3^ M), dexamethasone (1.0′10^−8^ M), dexamethasone (0.1 *μ*M), 10 *μ*M *β*-glycero-phosphate, and ascorbate (50 *μ*M) for 21 weeks. To assess mineralization, the cultures were stained with Alizarian Red S.

### 2.3. Immunophenotyping

The immunophenotypic features of the isolated cells were confirmed by flow cytometry. Briefly, the cells were dissociated with trypsin/EDTA; then, cell suspensions were stained using various antibodies against MSC markers including CD105-FITC (R&D Systems), CD90-PE (Dako), CD73-PE (Abcam), CD45-FITC (Dako), and CD34-PE (Biosciences) and secondary goat antimouse IgG-PE. The given cells were also labeled with isotype-matched antibodies and subsequently served as background controls. Finally, the cells were treated with suitable secondary antibodies, and the samples were submitted to the FACSCalibur cytometer (Becton Dickinson). Data analysis was performed using the CELL QUEST software.

### 2.4. Cell Transfection

For the purpose of transient transfections, the hBMSCs were seeded in 6-well plates the day before transfection. The miRNA-183, -96, and -182 and the scramble were also transfected into confluent (∼60%) cells at a concentration of 50 nM using Lipofectamine (Invitrogen's Lipofectamine 2000) according to the manufacturer's instructions. The evaluation of cell transfection was also carried out via qRT-PCR in 24 and 48 hours after transfection. It should be noted that each transfection was performed in triplicate.

### 2.5. qRT-PCR for Detection of MiRNA-96, -182, and -183 Expression Levels

The qRT-PCR was used to determine the expression of miRNA-183, -96, and -182 in the hBMSCs after transfection. The RNA extraction was then carried out using miRCURY RNA isolation kit (Exiqon, Denmark) for 24 and 48 hours following cell transfection. Universal cDNA synthesis kit (Exiqon, Denmark) was similarly used for the synthesis of cDNA. The qRT-PCR was also performed via SYBR green master mix kit (Exiqon, Denmark), and specific primers were determined for miRNA-183, -96, and -182 (Exiqon, Denmark) Table 1. In addition, snord was employed as the internal control and the 2^−ΔΔCt^ method was used for data analysis in the qRT-PCR test. The qRT-PCR was then performed using the Rotor-Gene 6000 (Corbett, Australia) with the following thermal cycling profile: 95°C for 2 minutes, followed by 40 cycles of amplification (95°C for 5 seconds, 60°C for 30 seconds).

### 2.6. qRT-PCR for Gene Expression Evaluation

Total RNA extract using TRIzol (Invitrogen), according to the manufacturer's instructions, and then the single-stranded cDNA were synthesized with cDNA Reverse Transcription Kit (Takara Shuzo, Otsu, Japan). Subsequently, the quantification of mRNAs levels was performed using a Rotor-Gene 6000 real-time PCR cycler (Corbett, Australia) via SYBR® Green PCR Master Mix. The following thermal settings were also used: 95°C for 5 minutes followed by 40 cycles of 95°C for 15 seconds and 72°C for 25 seconds. The primers used for each studied gene are presented in Table 2. The relative expression levels of the target genes were also normalized to GAPDH and then the data were analyzed using the 2^−∆∆Ct^ relative expression method.

### 2.7. Immunofluorescent Analysis

Immunofluorescent staining was employed for the hBMSCs after 24 and 48 hours of transient transfections. The cells were then fixed by 4% formaldehyde and permeabilized with 0.5% Triton X-100. After that, the cells were reacted with primary antibodies for RHO (1 : 500; Santa Cruz Biotechnology), CRX (1 : 500; Santa Cruz Biotechnology), at 4°C for 24 hours, and subsequently reacted with secondary antibody, goat antimouse IgG H&L (1 *μ*g/ml; Abcam, Cambridge, MA.), at room temperature for 1 hour in a dark place at ambient temperature. In addition, the cells were incubated with 4′,6′-diamidino-2-phenylindole hydro-chloride (1DAPI; Sigma, the United States) for 30 seconds. Finally, the preparations were examined under a fluorescence microscope (Olympus Corp., Tokyo, Japan). Ultimately, control experiments were conducted.

### 2.8. Statistical Analysis

All the data were obtained from more than three independent experiments and then analyzed using Graph Pad software (San Diego, California, USA). Unpaired Student *t*-test and one-way analysis of variance (ANOVA) or Kruskal–Wallis test followed by Dunnett's test were also used according to the data sets. *p* values lower than 0.05 were considered significant.

## 3. Results

### 3.1. Isolation and Identification of MSCs from Bone Marrow and Differentiation of hBMSCs

**Figure 1:**
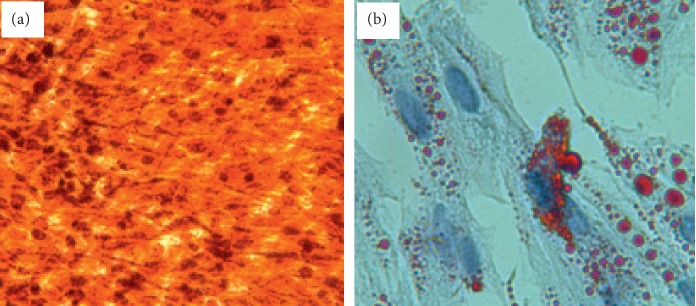
Differentiation potential of BMSCs. (a, b) Results of Alizarin Red S and Oil Red O staining showing osteocytes and adipocytes differentiation of BMSCs, respectively.

The MSCs were isolated from human bone marrow. First, the isolated cells as MSCs based on their morphology, their capacity to proliferate extensively, and their culture-adherent criterion in growth medium were studied in vitro. Then, the identity of the MSCs was confirmed based on their multipotent differentiation potential by their culturing in induction medium. MSCs were also differentiated to adipogenic and osteogenic lineages. Moreover, Alizarin Red S for osteocyte (Figure 1(a)) and Oil Red O staining for adipocyte (Figure 1(b)) were reported positive.

### 3.2. Phenotypic Analysis of hBMSCs Specific Markers

**Figure 2:**
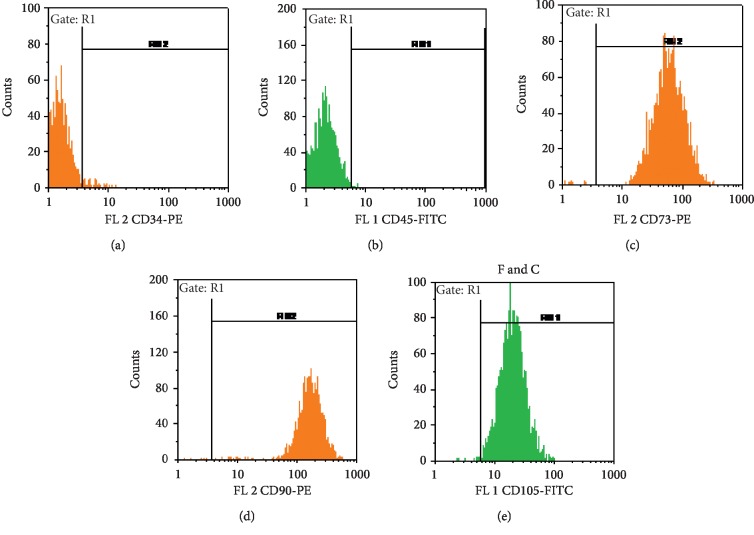
Flow cytometry analysis of BMSCs: BMSCs (two passage cells) were analyzed via fluorescence-activated cell sorting and Cell Quest software for expression of CD34 (a), CD45 (b), CD73 (c), CD90 (d), and CD105 (e).

In order to prove the stemness of the BMSCs derived from human bone marrow, the cells were analyzed against specific BMSCs antibodies and flow cytometric analysis showed positive morphological features as well as qualitative properties of the BMSCs. The population of BMSCs displayed positive expression of CD34 marker (Figure 2). Among CD34 positive cells, 71.4% of the cells expressed CD133, 89.9% of them expressed CD309, and 93.9% of such cells expressed CD117 marker. The mean percentage of CD expression of the three antigens in 5 different passages was compared using repeated measures ANOVA. The results demonstrated no significant difference in their expression. These cells were also stained positively for CD44 and CD13, but they were negative or very low positive for CD34.

**Figure 3:**
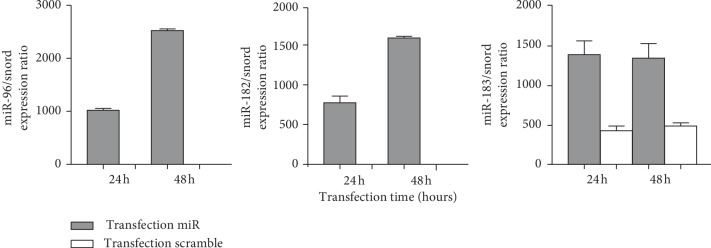
MiRNA-96, -182, and -183 expression in hBMSCs transfected with miRNA-96, -182, and -183 and scramble. The qRT-PCR showed significant upregulation of miRNA-96, -182, and -183 in transfected cells after 24 and 48 hours (*p* < 0.001).

Expression levels of miRNA-96, -182, and -183 in hBMSCs transfected with miRNA-96, -182, and -183 and scramble were analyzed using qRT-PCR (Figure 3). As shown in Figure 3, the miRNA-96, -182, and -183 was highly expressed in hBMSCs transfected with miRNA-96, -182, and -183 compared with those transfected with scramble (*p* < 0.001).

### 3.3. MiR-183/96/182 Targeting Multiple Retina Development-Related Genes

**Figure 4:**
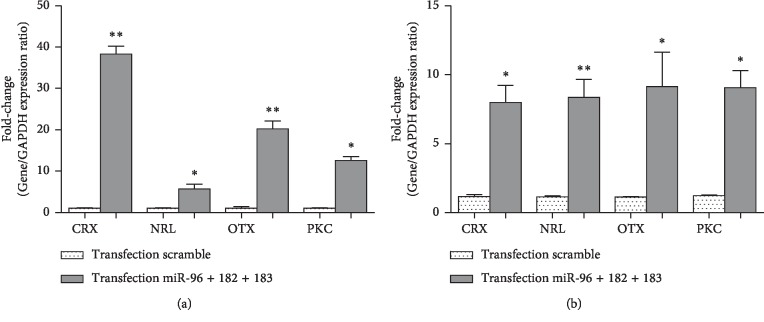
Evaluation of the expression of CRX, NRL, OTX2, and PKC*α* in hBMSCs transfected with miR-183/96/182 mimics and scramble. (a) The qRT-PCR showed significant upregulation of CRX, NRL, OTX2, and PKC*α* mRNAs in the transfected cells after 24 hours. (b) The qRT-PCR revealed significant upregulation of CRX, NRL, OTX2, and PKC mRNAs in the transfected cells after 48 hours. The data were then normalized to GAPDH. All the data represented the mean ± standard deviation (SD) of the three independent experiments with *n* = 3. Asterisks showed significance expression rate: ^*∗*^*p* < 0.05^*∗∗*^*p* < 0.001.

**Figure 5:**
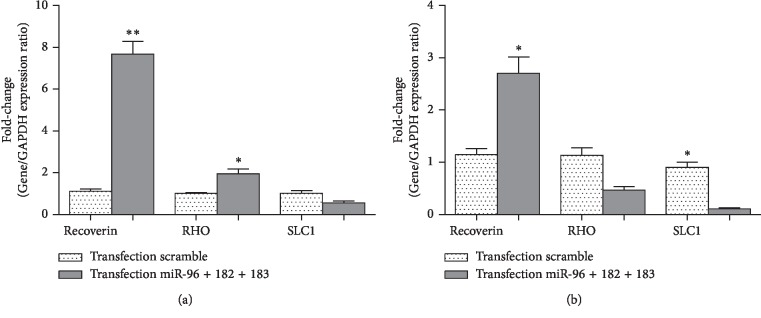
Evaluation of the expression of recoverin, RHO, and SLC1A1 in hBMSCs transfected with miR-183/96/182 mimics and scramble. (a) The qRT-PCR showed significant upregulation of recoverin and RHO mRNAs in the transfected cells after 24 hours. (b) The qRT-PCR revealed significant upregulation of recoverin mRNAs in the transfected cells after 48 hours. However, SLC1A1 did not lead to any significant differences in expression between transfected cells with miR-183/96/182 compared to transfected cells with scramble after 24 and 48 hours. The data were then normalized to GAPDH. All the data represented the mean ± standard deviation (SD) of the three independent experiments with *n* = 3.

It was hypothesized that miRNAs could target multiple retina development-related genes and it was also expressed at a higher level in retina than in somatic stem cells. To investigate this hypothesis, miR-183/96/182 mimics in the hBMSCs were transfected to induce neural retina differentiation of MSCs. The degree of miRNA overexpression was similarly monitored using qRT-PCR after transfection of miR-183/96/182 mimics into hBMSCs at 50 nM. Mature levels of miR-183/96/182 mimics were further elevated relative to control-treated cells, 24 and 48 hours following transfection. In this respect, retina development-related transcription factors such as CRX, OTX2, NRL, and PKC*α* as well as photoreceptor differentiation marker (recoverin and RHO) were detected using qRT-PCR method in transfected cells. Gene expression levels were also quantitatively assessed in these cells, and it was observed that the expression levels of CRX, OTX2, NRL, and PKC-*α* had increased by 38.1, 20.3, 5.5, and 12.2 folds, respectively, after 24 hours (Figure 4(a)) and had been also added by 7.6, 9.24, 8.24, and 8.92 folds, respectively, after 48 hours (Figure 4(b)). These data indicated that miR-183/96/182 mimics could have induced expressions of several retina development-related genes including CRX, OTX, NRL, and PKC-*α* following transfections which could also induce expressions of photoreceptor differentiation marker (recoverin and RHO) after 24 and 48 hours (Figures 5(a) and 5(b)). It was found that modulation of miR-183/96/182 mimic levels did not alter the proliferation or the morphology of BMSCs after 24 and 48 hours of transfection. Taken together, the results suggested that miR-183/96/182 was a positive regulator of photoreceptor-like cell differentiation of the hBMSCs.

### 3.4. Expression of Photoreceptor Cell Markers in Induced hBMSCs Using MiR-183/96/182

**Figure 6:**
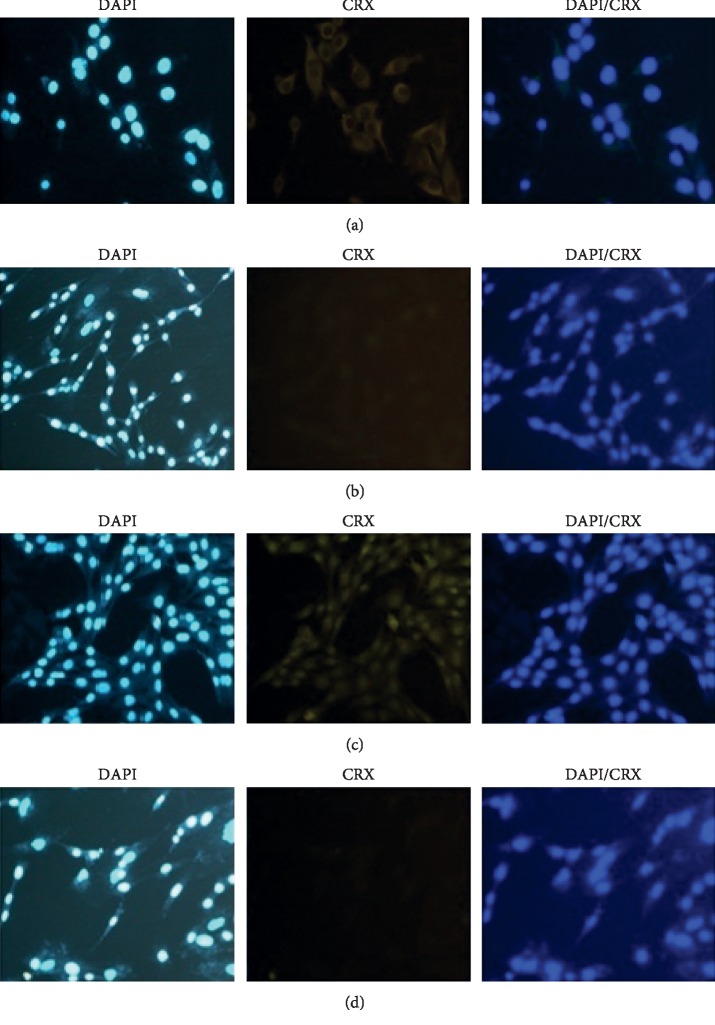
The hBMSCs that overexpressed miR-183/182/96 led to photoreceptor-like cells. (a, b) Expression of CRX in hBMSCs transfected with miR-183/96/182 and scramble, respectively, after 24 hours. (c, d) Expression of CRX in hBMSCs transfected with miR-183/96/182 and scramble, respectively, after 48 hours. Scale bars were 25 mm.

**Figure 7:**
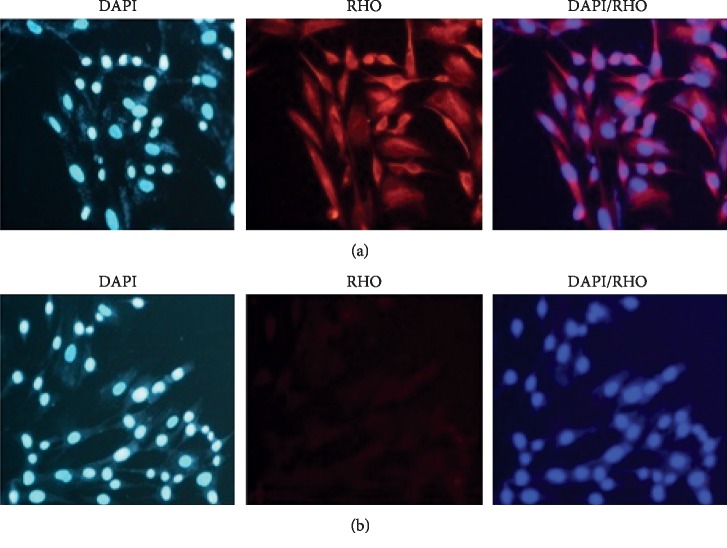
The hBMSCs that overexpressed miR-183/96/182 led to photoreceptor-like cells. (a, b) Expression of RHO in hBMSCs transfected with miR-183/96/182 and scramble, respectively, after 24 hours. Scale bars were 25 mm.

To further validate the differentiation potential of the induced hBMSCs, the expression of photoreceptor genes such as CRX and RHO was visualized with immunocytochemistry technique after transfecting the miR-183/96/182 mimics into the hBMSCs. The induced hBMSCs were also observed with a fluorescence microscope (Olympus, Tokyo, Japan) and at 5x and 2x magnifications. The cell nuclei were also stained with 4, 6-diamidino-2-phenylindole staining (DAPI). Accordingly, immunofluorescence staining showed that the CRXs had been expressed positively in the induced hBMSCs 24 and 48 hours after miR-183/96/182 mimics transfection (Figures 6(a) and 6(c)), compared with the control group (Figures 6(b) and 6(d)). The expression of RHO was also analyzed and IF staining showed that the expression of the RHO had been positively reported in the induced cells compared with the control group after 24 hours (Figures 7(a) and 7(b)).

## 4. Discussion

In this study, miR-183 cluster was transfected into the hBMSCs. The results showed that increased expression levels of miR-183 cluster could lead to a rising trend in the expression of some photoreceptor markers. These genes also had a key role in the differentiation of photoreceptors. It should be noted that hBMSCs have shown high potentials of transdifferentiation into a variety of neural cell types in vitro [1]. Therefore, they are considered as worthy candidates within reprogramming studies and cell therapy approaches [3]. Significant overexpression of OTX2, CRX, NRL, and PKC genes were also detected after 24 and 48 hours after mimic-miR-183 transfection probably resulted from alternations in expression profile of the hBMSCs influenced by ectopic overexpression of miR-183 cluster. These findings suggested that miR-183 (183/96/182) could play a pivotal role in neural retina in vivo via positive regulation of neural retina differentiation into the hBMSCs leading to reduced ectopic bone formation. Moreover, the CRX showed a significant growth in mRNA and protein levels in the transfected group with miR-183 (183/96/182) compared to the control group. It should be noted that OTX2 and CRX are considered as key regulators in postmitotic photoreceptor precursors and also play important roles in regulating a wide range of photoreceptor-specific genes that are critically significant for cone and rod genes [22]. Furthermore, they are essential for photoreceptor morphogenesis, terminal differentiation of photoreceptor cells, synaptic terminals, and phototransduction pathways. Overexpression of OTX2-inducted retinal transdifferentiation of human dermal fibroblast cells has been also reported [13]. The present findings and those in recent studies confirmed that the OTX family transcription factor was essential for the differentiation of late-born retinal neurons [22]. Moreover, the qRT-PCR analysis demonstrated an increase in one other marker of photoreceptor NRL, known as a transcription factor, recognized as a critical factor inducing rod photoreceptor-specific genes, cooperating with CRX and NR2E3, and promoting rod photoreceptor fate [23–25]. The NRL gene knockdown also resulted in the development of defect rods and converted it into the S opsin expressing cones [23]. PKC was also identified as another marker in rod bipolar cells whose mRNA levels had significantly increased, 24 and 48 hours following transfection of miR-183 cluster. It should be noted that PKC complex is required for regulation of polarity in postmitotic photoreceptors as well as proper laminar formation of the entire retina [26]. Moreover, PKC knockout could induce impaired development of photoreceptor and inactivated neuronal connections [26]. By the end of the differentiation, neural retina cell markers, recoverin, and RHO were highly expressed 24 hours following transfection, but RHO mRNA levels had significantly reduced after 48 hours. It should be noted that Slc1A1 is considered as one of the targets of miR-183/96/182 cluster existing in rods. It is also known as a voltage-dependent glutamate transporter that helps SLC1A7 remove glutamate (neurotransmitter) from synaptic cleft in rods and cones [27]. Slc1A1 expression also increases in darkness while miR-183/96/182 is downregulated during dark conditions. In fact, SLC1A1 overexpression is accompanied by downregulating of miR183/96/182 in dark conditions [27]. The present findings revealed that miR-183 cluster targeting the SLC1A1 had downregulated it. Therefore, the results of this study revealed that the hBMSCs had the potential to differentiate into photoreceptor-like cells with the help of miR-183/96/182 in suitable conditions. The hBMSCs could also differentiate into photoreceptor cells provided that there were specific conditions for differentiation into culture medium. Using retrovirus vector for overexpression of CRX, OTX2 in fibroblast cells could also lead to the apoptosis of the cells. It should be noted that viral vectors have toxic effects in gene therapy and miRNAs have good potentials to replace viral vectors [28, 29]. A number of miRNAs are thus essential for the formation and survival of photoreceptor cells whose downregulation can result in blindness. In the present study, the miR-183 cluster as a positive regulator of the hBMSCs photoreceptor differentiation was identified. The data obtained from in vitro experiments also revealed that overexpression of the miR-183 cluster functioning had enhanced photoreceptor differentiation of the hBMSCs, whereas previous studies had demonstrated that the downregulation of the expression of the miR-183 cluster had resulted in retinal degenerations and malignancy in different animal models [27]. In a study, a miR-182 KO mouse line was produced, which did not show significant changes in their retinal structure. This is the first loss of function study of a miRNA extensively expressed in the retina. The miR-182 likely is not a major determinant of retinal development, maintenance, or survival of cells in the retina. This study was the first step in clarifying the role of miRNAs in the retina [30]. But recently in a study, results revealed a relationship between miR-182 and retinal development, suggesting that miR-182 may play a vital role in maintaining retinal function, the findings showed that miR-182 is primarily a key to maintaining retinal function, which highlights the important role of miR-182 in the retina of the mammalian [31]. In another study, ectopic overexpression of miR-183 cluster in human retinal pigment epithelial cells (hRPE) could lead to reprogramming of hRPE cells and trigger neuronal cell fate [32]. Accordingly, the miR-183 cluster can play an important role in development and normal functioning of sensory organs [32], as expressed in rods, cones, and bipolar cells. It may also play critical roles in differentiation, survival, and suitable functioning of retinal neurons especially photoreceptors [32]. Taken together, it was concluded that the overexpression of the miR-183 cluster could induce in vitro transdifferentiation of the hBMSCs into photoreceptor neuron-like cells via increasing the expressions of neuroretinal progenitors and terminal differentiation markers. Therefore, it was assumed that miRNA as a therapeutic strategy could help in stem cell-based therapies for retinal degenerative diseases.

## Figures and Tables

**Table 1:** MiRNA sequences in the study (adapted from [21]).

miRBase accession	miRNA name	miRNA sequence (5′ ⟶ 3′)
MIMAT0000095	miR-96	UUUGGCACUAGCACAUUUUUGCU
MIMAT0000259	miR-182	UUUGGCAAUGGUAGAACUCACACU
MIMAT0000261	miR-183	UAUGGCACUGGUAGAAUUCACU

**Table 2:** Primers used in qPCR for mRNAs.

Name		Sequence
CRX		F-GGAGCTGGAGGCACTGT
		R-ACAAACCTGAACCCTGGAC
OTX2		F-CATGAGGCTGTAAGTTCCAC
		R-TTGTTTGGAGGTGCAAAGTC
PKC		F-GATCGCCACCTACCGCAA
		R-CCTCAGGCACAGTCGTCT
NRL		F-CCAGAGGAGACAGGAGCC
		R-TCCCGCACAGACATCGAGA
SLC1A1		F-CATCAGTATCACGGCCACA
		R-GGCACTCAGCACAATCACCA
RCVRN		F-GGGACCATCAGCAAGAAT
		R-GATCTTCTCGGCTCGCTTT
RHO		F-GTCCAGGTACATCCCCG
		R-ACGAACATGTAGATGACAA
GAPDH		F-GAGTCCACTGGCGTCTTCAC
		R-ATGACGAACATGGGGGCATC

## Data Availability

The data used to support the findings of this study are available from the corresponding author upon request.

## References

[1] Kicic A., Shen W.-Y., Wilson A. S., Constable I. J., Robertson T., Rakoczy P. E. (2003). Differentiation of marrow stromal cells into photoreceptors in the rat eye. *The Journal of Neuroscience*.

[2] Ramsden C. M., Powner M. B., Carr A.-J. F., Smart M. J. K., da Cruz L., Coffey P. J. (2013). Stem cells in retinal regeneration: past, present and future. *Development*.

[3] Smith L. E. H. (2004). Bone marrow-derived stem cells preserve cone vision in retinitis pigmentosa. *Journal of Clinical Investigation*.

[4] Garcia J. M., Mendonca L., Brant R., Abud M., Regatieri C., Diniz B. (2015). Stem cell therapy for retinal diseases. *World Journal of Stem Cells*.

[5] Ng T. K., Fortino V. R., Pelaez D., Cheung H. S. (2014). Progress of mesenchymal stem cell therapy for neural and retinal diseases. *World Journal of Stem Cells*.

[6] Mathivanan I., Trepp C., Brunold C., Baerlocher G., Enzmann V. (2015). Retinal differentiation of human bone marrow-derived stem cells by co-culture with retinal pigment epithelium in vitro. *Experimental Cell Research*.

[7] Zhang M., Zhang F., Sun J. (2017). The condition medium of mesenchymal stem cells promotes proliferation, adhesion and neuronal differentiation of retinal progenitor cells. *Neuroscience Letters*.

[8] Soleimannejad M., Ebrahimi-Barough S., Nadri S. (2017). Retina tissue engineering by conjunctiva mesenchymal stem cells encapsulated in fibrin gel: hypotheses on novel approach to retinal diseases treatment. *Medical Hypotheses*.

[9] Qiang S., Alsaeedi H. A., Yuhong C. (2018). Morphological and genetical changes of endothelial progenitor cells after in—vitro conversion into photoreceptors. *Journal of Photochemistry and Photobiology B: Biology*.

[10] Lee H.-S., Huang G.-T., Chiang H. (2003). Multipotential mesenchymal stem cells from femoral bone marrow near the site of osteonecrosis. *Stem Cells*.

[11] Choi S. W., Shin J. H., Kim J. J. (2016). Direct cell fate conversion of human somatic stem cells into cone and rod photoreceptor-like cells by inhibition of microRNA-203. *Oncotarget*.

[12] Yuan Z., Ding S., Yan M. (2015). Variability of miRNA expression during the differentiation of human embryonic stem cells into retinal pigment epithelial cells. *Gene*.

[13] Ohana R., Weiman-Kelman B., Raviv S. (2015). MicroRNAs are essential for differentiation of the retinal pigmented epithelium and maturation of adjacent photoreceptors. *Development*.

[14] Yoo A. S., Sun A. X., Li L. (2011). MicroRNA-mediated conversion of human fibroblasts to neurons. *Nature*.

[15] Hu G., Huang K., Yu J. (2012). Identification of miRNA signatures during the differentiation of hESCs into retinal pigment epithelial cells. *PloS One*.

[16] Lumayag S., Haldin C. E., Corbett N. J. (2013). Inactivation of the microRNA-183/96/182 cluster results in syndromic retinal degeneration. *Proceedings of the National Academy of Sciences*.

[17] Xu S. (2009). microRNA expression in the eyes and their significance in relation to functions. *Progress in Retinal and Eye Research*.

[18] Xiang L., Chen X.-J., Wu K.-C. (2017). miR-183/96 plays a pivotal regulatory role in mouse photoreceptor maturation and maintenance. *Proceedings of the National Academy of Sciences*.

[19] Fan J., Jia L., Li Y. (2017). Maturation arrest in early postnatal sensory receptors by deletion of the miR-183/96/182 cluster in mouse. *Proceedings of the National Academy of Sciences*.

[20] Busskamp V., Krol J., Nelidova D. (2014). miRNAs 182 and 183 are necessary to maintain adult cone photoreceptor outer segments and visual function. *Neuron*.

[21] Mahmoudian-Sani M.-R., Jami M.-S., Mahdavinezhad A., Amini R., Farnoosh G., Saidijam M. (2018). The effect of the microrna-183 family on hair cell-specific markers of human bone marrow-derived mesenchymal stem cells. *Audiology and Neurotology*.

[22] Terrell D., Xie B., Workman M. (2012). OTX2 and CRX rescue overlapping and photoreceptor-specific functions in the Drosophila eye. *Developmental Dynamics*.

[23] Sanuki R., Omori Y., Koike C., Sato S., Furukawa T. (2010). Panky, a novel photoreceptor-specific ankyrin repeat protein, is a transcriptional cofactor that suppresses CRX-regulated photoreceptor genes. *FEBS Letters*.

[24] Tran N. M., Zhang A., Zhang X., Huecker J. B., Hennig A. K., Chen S. (2014). Mechanistically distinct mouse models for CRX-associated retinopathy. *PLoS Genetics*.

[25] Peng G.-H., Chen S. (2011). Active opsin loci adopt intrachromosomal loops that depend on the photoreceptor transcription factor network. *Proceedings of the National Academy of Sciences*.

[26] Koike C., Nishida A., Akimoto K. (2005). Function of atypical protein kinase C in differentiating photoreceptors is required for proper lamination of mouse retina. *Journal of Neuroscience*.

[27] Krol J., Busskamp V., Markiewicz I. (2010). Characterizing light-regulated retinal microRNAs reveals rapid turnover as a common property of neuronal microRNAs. *Cell*.

[28] Seko Y., Azuma N., Kaneda M. (2012). Derivation of human differential photoreceptor-like cells from the iris by defined combinations of CRX, RX and NEUROD. *PloS One*.

[29] Seko Y., Azuma N., Ishii T. (2014). Derivation of human differential photoreceptor cells from adult human dermal fibroblasts by defined combinations ofCRX, RAX, OTX2 and NEUROD. *Genes to Cells*.

[30] Jin Z. B., Hirokawa G., Gui L. (2009). Targeted deletion of miR-182, an abundant retinal microRNA. *Molecular Vision*.

[31] Wu K.-C., Chen X.-J., Jin G.-H. (2019). Deletion of miR-182 leads to retinal dysfunction in mice. *Investigative Opthalmology & Visual Science*.

[32] Davari M., Soheili Z.-S., Samiei S., Sharifi Z., Pirmardan E. R. (2017). Overexpression of miR-183/-96/-182 triggers neuronal cell fate in Human Retinal Pigment Epithelial (hRPE) cells in culture. *Biochemical and Biophysical Research Communications*.

